# Identification of a novel S6K1 inhibitor, rosmarinic acid methyl ester, for treating cisplatin-resistant cervical cancer

**DOI:** 10.1186/s12885-019-5997-2

**Published:** 2019-08-06

**Authors:** Ki Hong Nam, Sang Ah Yi, Gibeom Nam, Jae Sung Noh, Jong Woo Park, Min Gyu Lee, Jee Hun Park, Hwamok Oh, Jieun Lee, Kang Ro Lee, Hyun-Ju Park, Jaecheol Lee, Jeung-Whan Han

**Affiliations:** School of Pharmacy, Sungkyunkwan University, Suwon, 16419 Republic of Korea

**Keywords:** Rosmarinic acid methyl ester, S6K1, Autophagy, Apoptosis, Cervical cancer, Cisplatin resistance

## Abstract

**Background:**

The mTOR/S6K1 signaling pathway is often activated in cervical cancer, and thus considered a molecular target for cervical cancer therapies. Inhibiting mTOR is cytotoxic to cervical cancer cells and creates a synergistic anti-tumor effect with conventional chemotherapy agents. In this study, we identified a novel S6K1 inhibitor, rosmarinic acid methyl ester (RAME) for the use of therapeutic agent against cervical cancer.

**Methods:**

Combined structure- and ligand-based virtual screening was employed to identify novel S6K1 inhibitors among the in house natural product library. In vitro kinase assay and immunoblot assay was used to examine the effects of RAME on S6K1 signaling pathway. Lipidation of LC3 and mRNA levels of ATG genes were observed to investigate RAME-mediated autophagy. PARP cleavage, mRNA levels of apoptotic genes, and cell survival was measured to examine RAME-mediated apoptosis.

**Results:**

RAME was identified as a novel S6K1 inhibitor through the virtual screening. RAME, not rosmarinic acid, effectively reduced mTOR-mediated S6K1 activation and the kinase activity of S6K1 by blocking the interaction between S6K1 and mTOR. Treatment of cervical cancer cells with RAME promoted autophagy and apoptosis, decreasing cell survival rate. Furthermore, we observed that combination treatment with RAME and cisplatin greatly enhanced the anti-tumor effect in cisplatin-resistant cervical cancer cells, which was likely due to mTOR/S6K1 inhibition-mediated autophagy and apoptosis.

**Conclusions:**

Our findings suggest that inhibition of S6K1 by RAME can induce autophagy and apoptosis in cervical cancer cells, and provide a potential option for cervical cancer treatment, particularly when combined with cisplatin.

**Electronic supplementary material:**

The online version of this article (10.1186/s12885-019-5997-2) contains supplementary material, which is available to authorized users.

## Background

Cervical cancer is one of the most common malignant gynaecological tumors and is primarily caused by persistent human papilloma virus (HPV) infection [1]. Although effective vaccines against high-risk HPV strains significantly lower the occurrence of cervical cancer, these vaccines have only prophylactic effects without therapeutic effects against HPV-infected lesions [2, 3]. The currently existing remedies for cervical cancer are surgery, chemoradiotherapy, or both; however, these options are limited in patients with metastatic or recurrent cervical cancers after platinum-based chemoradiotherapy [4–6]. Therefore, the development of targeted therapeutics utilizing pathological mechanisms is necessary to cure advanced or recurrent cervical cancer.

The HPV infection-mediated pathogenesis of cervical cancer is closely related to the activation of multiple intracellular signaling pathways [7, 8]. The mammalian target of rapamycin (mTOR) is one such signaling molecule that has been reported to be activated in cervical cancer [8–12]. Immunostaining analyses have shown that p-mTOR, p-p70S6K1, and p-S6 are highly detected in HPV-positive lesions and cervical cancer cell lines [9–12], and these contribute to the survival of cervical cancer cells [11]. Pharmaceutical inhibition of this signaling cascade in mice and cell lines effectively suppressed tumorigenesis, cell growth, and proliferation of cervical cancer cells [12–14]. These findings have demonstrated that the mTOR/S6K1 signaling pathway can be used as a prognostic marker or therapeutic target for cervical cancer treatment.

Cisplatin, a platinum-based drug, is a primary chemotherapeutic agent that is used in combination with radiotherapy to treat cervical cancer [15, 16]. Unfortunately, the frequent acquisition of resistance to cisplatin in cervical cancer patients is a major cause of therapeutic failure [17]. Among the multifactorial mechanisms underlying chemoresistance, overexpression or activation of the Akt/mTOR pathway critically contributes to cisplatin resistance by attenuating p53 activity [18, 19]. The majority of studies have suggested that co-treatment with an mTOR inhibitor including rapamycin greatly enhanced the therapeutic activity of cisplatin against several cisplatin resistant cell lines, causing activation of autophagy and subsequent apoptosis [9, 14, 19–24]. As the broad action of rapamycin can cause unexpected side effects, seeking more specific inhibitor is considered to be an effective way to overcome cisplatin resistance.

Here, we performed structure-based screening of single compound library and identified that rosmarinic acid methyl ester (RAME) is a potent inhibitor of the mTOR/S6K1 signaling pathway. RAME treatment of cervical cancer cells effectively inhibited activation of S6K1 as well as the kinase activity of S6K1. We also observed an increase in autophagy and apoptotic cell death after RAME treatment in cervical cancer cell lines. Moreover, co-treatment of RAME with cisplatin sensitized cisplatin-resistant cervical cancer cell line and synergistically caused the induction of autophagy and apoptosis. Collectively, our findings revealed that RAME, a natural-derived compound, is a candidate therapeutic substance for cervical cancer patients, particularly for those whose cancer displayed cisplatin resistance.

## Methods

### Reagents

Anti-p70 S6K1 (Santa Cruz Biotechnology, Dallas, TX; SC-230), anti-phospho (T389) p70 S6K1 (Cell Signaling Technology, Danvers, MA; #9205), anti-S6 (Cell Signaling Technology; #2217), anti-phospho (S235/236) S6 (Cell Signaling Technology; #4856), anti-GFP (Santa Cruz Biotechnology; SC-9996), anti-PARP-1 (Santa Cruz Biotechnology; SC-7150), anti-Akt1/2/3 (Santa Cruz Biotechnology; SC-8312), anti-phospho (S473) Akt (Santa Cruz biotechnology; SC-7958), anti-LC3B (Cell Signaling Technology; #2775), anti-p53 (Santa Cruz Biotechnology, Dallas; SC-126), and anti-actin (Millipore, Temecula, CA; mab1501) antibodies were utilized in this study.

### Cell culture

HeLa (ATCC® CCL-2), A549 (ATCC® CCL-185), H1299 (ATCC® CRL-5803) cells were obtained from the American Type Culture Collection (ATCC) and SiHa cells (ATCC® HTB-35) were generous gifts from Jung-Hye Choi (Kyung Hee University), who obtained the cells from ATCC. The cells were cultured as indicated in the instructions from ATCC and were grown under a fully humidified atmosphere of 95% air and 5% CO_2_ at 37 °C. Cells grown to 80–90% confluency was used for assays.

### Knockdown of S6K1

For the knockdown of S6K1, HeLa cells were transfected with siRNA targeting S6K1 using Lipofectamine 2000 reagent (Life Technologies) according to the manufacturer’s protocol. The siRNA sequences targeting S6K1 are as follow: forward, 5′-CACCCUUUCAUUGUGGACCUGAUUU-3′ and reverse, 5′-AAAUCAGGUCCACAAUGAAAGGGUG-3′.

### Virtual screening of natural product compound library

The docking screening was carried out using the Sybyl-X 2.1.1 package in Windows 7. The X-ray structure of the S6K1 kinase domain (PDB ID: 3WE4) [25] complexed with PF-470871 was downloaded from the RCSB Protein Data Bank (http:/www.rcsb.org/pdb/home/home.do). The structure was refined as follows: all water molecules were removed, the ligand was extracted, and the protein structure was optimized with the protein preparation module in Sybyl using the default parameters. The Surflex-Dock module embedded in Sybyl was used to conduct a docking screening of the in-house library containing 519 natural product compounds. The X-ray pose of bound ligand PF-470871 was assigned to generate the protomol, which defines the receptor’s binding cavity in which docked ligands are aligned. Protomol was generated with a threshold parameter of 0.50 and a bloat parameter of 0 Å. The main setting was 50 solutions per compound, and other parameters accepted the Surflex-Dock Geom default settings. The scoring function for Surflex-Dock is trained to estimate the dissociation constant (*K*_*d*_) expressed in –log *K*_*d*_ units. The final hitlist compounds were selected after evaluating for binding by combining the consensus scoring function CScore (consensus score > 3), Surflex-Dock total score (> 8), and Lipinski’s rule-of-five filter. Similarity-based virtual screening was conducted using flexible ligand superpositioning algorithm FlexS implanted in Sybyl [26]. The X-ray pose of PF-470871(PDB ID: 3WE4) was used as the template molecule. A higher similarity score represented a greater similarity of a tested molecule to the template molecule (maximum score is 10.0).

### Immunoblotting

The cells were lysed in Pro-Prep (iNtRON Biotechnology, Korea) and centrifuged at 13,000 rpm for 18 min. For immunoblotting, proteins of each sample were separated through SDS-polyacrylamide gel electrophoresis (PAGE). The proteins were transferred to polyvinylidene difluoride (PVDF) membranes with a semi-dry transfer apparatus (Bio-Rad, Hercules, CA). The membranes were incubated overnight with the indicated primary antibodies, then incubated with horseradish peroxidase-conjugated secondary antibodies for 1 h (Abcam). The signals were detected through chemiluminescence reagents (AbClon, Korea) and quantified with ImageJ program.

### Immunofluorescence

For the ectopic expression of the LC3B vectors, HeLa and SiHa cells were transfected with GFP-LC3B vectors using Lipofectamine 2000 reagent (Invitrogen, Carlsbad, CA), according to the manufacturer’s instructions. After 24 h, cells were fixed in 4$ paraformaldehyde and then GFP signal from ectopically expressed LC3B was observed using confocal microscope (Olympus FV-1000 confocal laser scanning microscope) with an Apochromat 60× objective.

### Quantitative real-time PCR (qPCR)

RNA extracts were prepared as previously described [27]. To extract total RNA, cells were lysed in Easy-Blue reagent (iNtRON Biotechnology). Then, 1 μg of total RNA was reversely transcribed into cDNA using a Reverse Transcription kit (Promega, USA). Quantitative real-time PCR was performed using KAPATM SYBR FAST qPCR (KAPABIOSYSTEMS) with the CFX96™ or Chromo4™ real-time PCR detector (Bio-Rad). The relative mRNA levels were normalised to the *GAPDH* mRNA levels for each reaction. The qPCR primer sequences used are as follow: *GAPDH* forward, 5′-GAGTCAACGGATTTGGTCGT-3′; *GAPDH* reverse, 5′-TTGATTTTGGAGGGATCTCG-3′; *ULK1* forward, 5′-GGACACCATCAGGCTCTTCC-3′; *ULK1* reverse, 5′-GAAGCC GAAGTCAGCGATCT-3′; *ATG5* forward, 5′-AGCAACTCTGGATGGGATTG-3′; *ATG5* reverse, 5′-CACTGCAGAGGTGTTTCCAA-3′; *BECN1* forward, 5′-AACCTCAGCCGAAGACTGAA-3′; *BECN1* reverse, 5′-GACGTTGAGCTGAGTGTCCA-3′; *ATG7* forward, 5′-ACCCAGAAGAAGCTGAACGA-3′; *ATG7* reverse, 5′-AGACAGAGGGCAGGATAGCA-3′; *ATG12* forward, 5′-GGCAGTAGAGCGAACACGAA-3′; *ATG12* reverse, 5′-GGGAAGGAGCAAAGGACTGA-3′; *ATG13* forward, 5′-CCCAGGACAGAAAGGACCTG-3′; *ATG13* reverse, 5′-AACCAATCTGAACCCGTTGG-3′; *Bax* forward, 5′-TCTACTTTGCCAGCAAACTGG-3′; *Bax* reverse, 5′-TGTCCAGCCCATGATGGTTCT-3′; *Noxa* forward, 5′-AGAGCTGGAAGTCGAGTGT-3′; *Noxa* reverse, 5′-GCACCTTCACATTCCTCTC-3′; *Puma* forward, 5′-GACCTCAACGCACAGTA-3′; *Puma* reverse, 5′-CTAATTGGGCTCCATCT-3′; *Gadd45α* forward, 5′-TGCGAGAACGACATCAACAT-3′; *Gadd45α* reverse, 5′-TCCCGGCAAAAACAAATAAG-3′; *p21* forward, 5′-CACCGAGACACCACTGGAGG-3′; *p21* reverse, 5′-GAGAAGATCAGCCGGCGTTT-3′; *14–3-3σ* forward, 5′-TTTCCTCTCCAGACTGACAAACTGTT-3′; *14–3-3σ* reverse, 5′-TAGAACTGAGCTGCAGCTGTAAA-3′.

### Cell viability assay

HeLa and SiHa cells were plated in 6 well plates at a density of 6 × 10^5^ and 2 × 10^5^ cells per well, respectively. Cells were treated with DMSO or RAME (40, 80 μM) for 24 and 48 h before the cells were counted. For cell counting, cells trypsinized using Trypsin EDTA were counted using a haemocytometer.

### In vitro kinase assay

In vitro kinase assay was performed as previously described [27]. Briefly, recombinant S6K1 (R&D systems, Minneapolis, MN; 896-KS), GST-S6 (Abnova, Taipei city, Taiwan; H00006194-P01), and H2B (BioLabs, MA, USA; M2505S) were used. The reactions were performed in the presence of 100 μM adenosine triphosphate (ATP) and kinase reaction buffer [25 mM Tris-HCl (pH 7.5), 5 mM β-glycerophosphate, 2 mM dithiothreitol (DTT), 0.1 mM Na_3_VO_4_, 10 mM MgCl_2_] at 37 °C for 45 min. The reactions were stopped with 5× Laemmli loading buffer and then subjected to immunoblot analysis.

### Clonogenic assay

For clonogenic assays, HeLa and SiHa cells were seeded in 1 × 10^3^ cells per well of a 6-well plate and cultured in complete media for 10~20 days. Cells were fixed with glutaraldehyde (6%), stained with 0.5% crystal violet, and photographed using a digital scanner. All experiments were performed at least three times. Representative experiments are shown.

### Statistical analysis

Statistical significance was analysed using the Student’s *t*-test (two-tailed) and assessed based on the *P*-value.

## Results

### RAME is identified as a novel S6K1 inhibitor by virtual screening of the natural product compound library

**Fig. 1:**
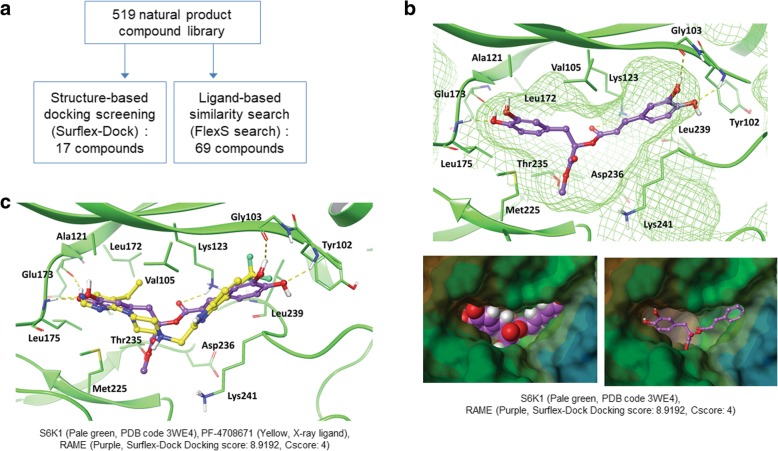
RAME, identified by virtual screening, is a novel S6K1 inhibitor. **a** Strategy for finding a novel S6K1 inhibitor by combining structure- and ligand-based virtual screening. **b** Docking model of RAME in the ATP-binding site of S6K1 (PDB id: 3WE4), which demonstrated a mesh (top) or MOLCAD lipophilic potential surface (bottom). The color of lipophilic potential ranges from brown (hydrophobic area) to green-blue (hydrophilic area). Carbon atoms are purple (RAME) and green (amino acid residues); nitrogen is blue; oxygen is red; hydrogen is grey. Hydrogen bonding interactions are represented by yellow dashes. **c** The docked pose of RAME overlays the X-ray pose of PF-4708671 (yellow carbon)

To identify novel S6K1 inhibitors, we conducted a virtual screening of the in-house library containing 519 compounds isolated from natural products. We employed both docking-based screening and a similarity-based search method to select the candidate compounds (Fig. 1a). First, Surflex-Dock docking was performed against the X-ray structure S6K1 kinase domain (PDB ID: 3WE4) and 17 candidate compounds were selected, considering their binding energy scores and drug-like properties (Table 1). Next, we used the FlexS program for flexible superpositioning of all the database compounds onto the rigid X-ray pose of PF-4708671 (PDB ID: 3WE4). From there, 69 compounds with similarity over 65% were selected (Table 2). The hit lists obtained from the two virtual screening methods were quite different and just two compounds (KR_CT_11 and RAME) were identified as high-ranking hits from both methods (Additional file: Figure S1). Then, we visually inspected the binding interactions between ligand and S6K1 kinase domain focusing on the hinge region, which is important for inhibitor activity. Only RAME (*R*-enantiomer) occupied the hinge region and formed hydrogen bonds with Glu173 and Leu175 (Fig. 1b), whereas KR_CT_11 did not fit into this region. As illustrated in Fig. 1b and c, the left-side catechol group faced the hinge region, and one OH group formed bidentate hydrogen bonds with the backbone carbonyl oxygen of Glu173 and the backbone amide NH of Leu175. The aromatic ring was surrounded by hydrophobic residues, such as Ala121, Leu172, and Met225, forming hydrophobic and van der Waals interactions. The methyl group of the methyl ester was involved in hydrophobic contact with the side chain of Met225, which could not be formed by RA. Two OH groups on the right-side catechol formed hydrogen bonds with Gly103 and Tyr102. In addition, the carbonyl oxygen atom of the central ester linker also formed a hydrogen bond with the side chain amine of Lys123. Overall, the docked pose of RAME appeared to be similar to the X-ray pose of PF-4708671 (Fig. 1c), but RAME formed more extensive polar interactions in the same active site of the S6K1 kinase domain [25]. These findings encouraged us to investigate the effects of RAME on S6K1 and its downstream signaling.

**Table 1:** Hit list 17 compounds selected by Surflex-Dock docking analysis

Selected 17 compounds (Total Score > 8 and C score > 3)												
Surflex-Dock Docking Results									Lipinski’s Properties			
Name	Total Score	Crash	Polar	D score	PMF score	G score	Chem score	C score	H-bond Acceptor	H-bond Donor	Molecular weight	cLogP
AMC8	8.3455	−2.1334	3.1423	− 150.1913	− 13.5711	−92.1301	− 23.2483	4	6	3	343.3737	2.2664
BBE4	8.0406	−1.684	5.2925	−118.3739	−9.5793	−205.3034	−22.8193	4	4	3	285.3377	1.782
JC24	8.6122	−2.588	4.8921	−142.4306	−49.9801	−227.2735	− 22.9057	4	8	4	390.3839	0.7192
JSYB21	9.122	−3.9792	4.8938	− 196.7893	9.5743	− 279.8751	−15.2224	4	13	6	478.4444	−2.0499
JSYB4 (RA)	8.4245	−2.2637	7.6215	−127.2991	−17.5161	− 184.1398	−20.575	4	8	5	360.3148	1.0996
KR_BK_10	8.0976	−0.9089	3.8661	−127.2817	−26.3626	− 220.0721	− 21.0439	5	7	4	378.4162	0.7522
KR_BK_16	8.4383	−0.6405	3.648	−123.7665	−14.3909	− 188.1732	−20.8374	5	6	3	360.401	1.6347
KR_BM_41	8.1458	−0.6331	5.9054	− 113.8569	−42.814	−115.0421	−23.1057	4	7	5	302.2357	1.5037
KR_CT_11	8.1698	−2.3809	1.0128	−148.0841	14.7862	− 253.8318	−18.5355	5	8	0	469.5268	3.0063
KR_HV_6	9.3254	−3.3264	4.8485	−153.1333	21.5821	−286.7884	−14.5041	4	10	6	416.4196	−1.1732
KR_HV_8	8.5056	−2.7215	4.3782	−155.9434	20.9705	− 231.0792	−15.0293	4	10	6	422.4673	−0.0712
KR_HV_9	9.3382	−2.5547	4.3424	− 152.7992	1.4948	− 265.2069	−12.6789	5	10	6	415.4117	−1.2102
KR_TR_6	8.3868	−1.8635	5.3265	−133.4614	−16.3225	−214.3578	−26.3579	5	5	3	313.3478	2.4172
SKB54	8.1219	−4.7382	6.1257	−206.3804	−25.3407	− 297.2112	−12.7596	5	12	8	448.4184	−2.2472
SRE10 (RAME)	8.9192	−1.7056	5.7246	− 138.018	−15.4552	−206.3927	−20.5586	4	8	4	374.3414	1.3942
TBDE6	8.0693	−1.0091	7.043	−112.1842	−36.4047	−40.4333	−26.3986	4	7	5	302.2357	1.5037
WBCC44	8.9582	−3.684	3.125	−178.3426	19.0338	− 289.5232	− 21.5875	4	11	4	466.4352	0.3119

**Table 2:** List of compounds with FlexS similarity score higher than 6.5. (The score of template molecule PF-4708671 = 10)

1	BBC32	7.7308	21	KR_CW_4	7.3366	41	KR_CT_1	6.9312	61	JC32	6.6894
2	TOH27	7.6826	22	LY2584702	7.2976	42	TOH30	6.9239	62	KR_PC_19	6.6871
3	KR_GE_56	7.6669	23	KR_CW_3	7.2956	43	JGCC121	6.9237	63	SRE10	6.6444
4	KR_PK_25	7.6421	24	TOH37	7.2726	44	DG2	6.9233	64	BSCC31	6.6342
5	KR_CW_7	7.6234	25	KR_PC_1	7.2496	45	TOH25	6.9217	65	BSCC6	6.6272
6	KR_PK_18	7.621	26	JC1	7.2219	46	KR_CW_1	6.898	66	PMBC2	6.6152
7	BBH3	7.6062	27	KR_PK_2	7.2159	47	KR_PC_12	6.8931	67	KR_BK_30	6.576
8	BBC7	7.6062	28	PFE5	7.2075	48	KR_CT_2	6.8671	68	KR_BK_13	6.5618
9	KR_GE_52	7.5701	29	KR_CT_13	7.1872	49	KR_CT_4	6.8218	69	KR_HV_11	6.5519
10	BBC33	7.503	30	KR_CW_2	7.1569	50	KR_CT_5	6.8207			
11	KR_CT_3	7.4814	31	KR_CW_9	7.1418	51	KR_CT_10	6.8103			
12	KR_CT_12	7.471	32	KR_PK_15	7.0917	52	Pfizer	6.7808			
13	KR_PK_23	7.4528	33	BKHC1	7.0725	53	Lilly	6.772			
14	JC8	7.4419	34	KR_CT_11	7.0143	54	JC12	6.7711			
15	KR_CT_7	7.3919	35	KR_LA_1	6.9976	55	KR_CT3	6.7694			
16	KR_CW_6	7.3867	36	KR_PK_19	6.9908	56	KR_HV_12	6.7583			
17	JGCC60	7.3785	37	JSY1	6.9793	57	KR_CT_8	6.736			
18	KR_CT2	7.374	38	KR_PGA_3	6.9675	58	KR_PN_4	6.7145			
19	KR_GE_53	7.3672	39	5,559,274	6.959	59	KR_BM_48	6.7062			
20	KR_PK_16	7.3587	40	KR_PGA_2	6.9566	60	BSCC7_	6.7011			

### RAME, not RA, inhibits the phosphorylation of S6 by S6K1

**Fig. 2:**
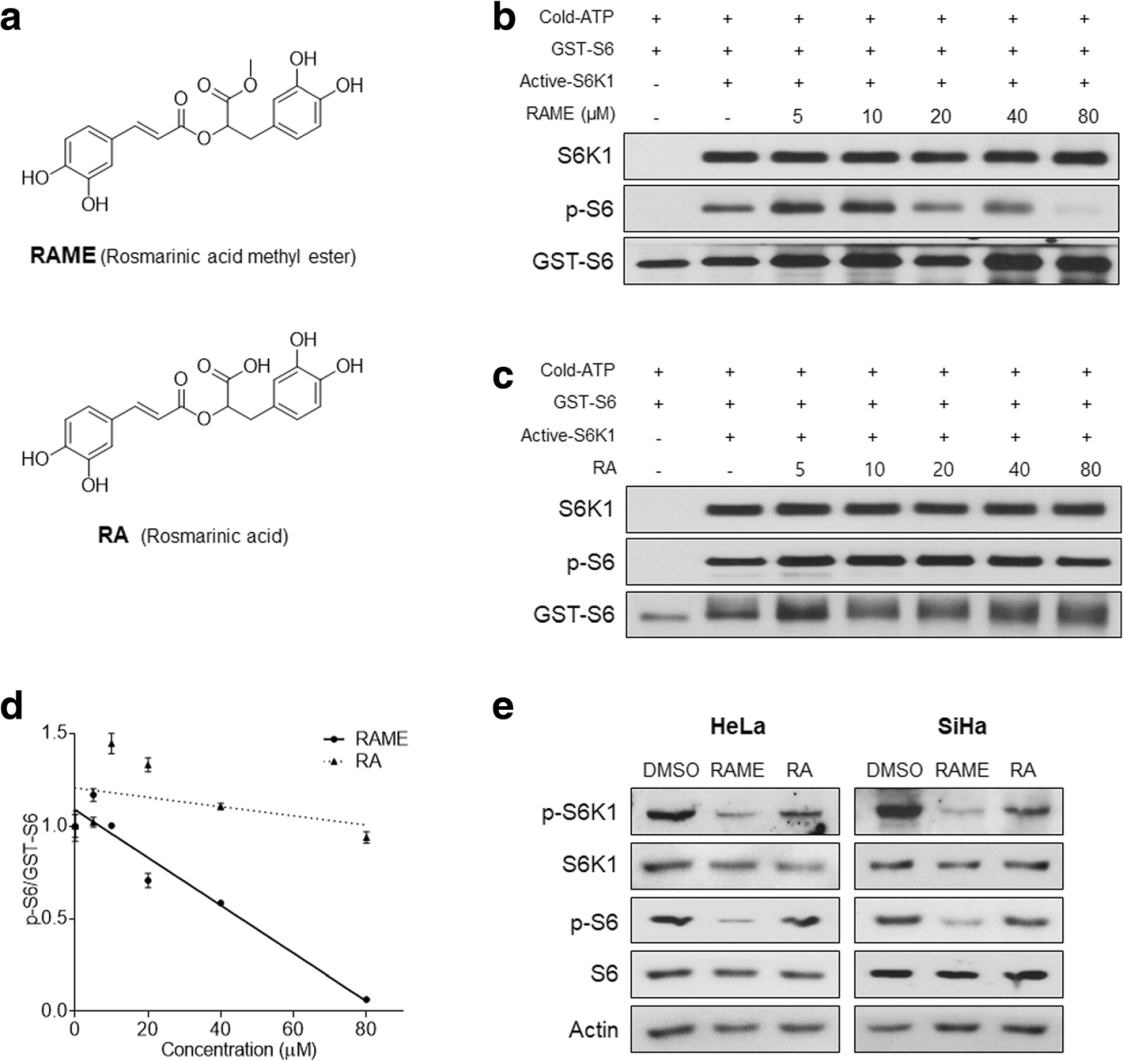
RAME, not RA, inhibits kinase activity of S6K1 in vitro and in vivo. **a** Structures of RAME and RA. **b** and **c** The in vitro kinase assay with RAME (**b**) or RA (**c**) was performed in a dose dependent manner using recombinant GST-S6, active S6K1, and cold-ATP. **d** Quantitative graph of (**b** and **c**). **e** Immunoblotting analysis of HeLa (left) and SiHa (right) cells treated with RAME (80 μM) or RA (80 μM) for 24 h

Based on the binding pose of RAME, we decided to evaluate the regulatory activity of RAME, compared with its parent compound rosmarinic acid (RA) (Fig. 2a). To evaluate whether RAME and RA affect the kinase activity of S6K1 in vitro, we conducted an in vitro kinase assay using recombinant S6K1 with GST-S6 protein as a substrate. RAME inhibited the phosphorylation of S6 in dose-dependent manner (Fig. 2b), but RA did not affect the S6K1-mediated phosphorylation of S6 (Fig. 2c, d). The phosphorylation of H2B S36, another representative S6K1 target [27], was also inhibited by incubation with RAME, as observed by in vitro kinase assay with recombinant H2B protein (Additional file 1: Figure S2). Next, we examined whether RAME and RA inhibit S6K1 activity also in vivo, by treating cervical cancer cell lines with RAME and RA (80 μM) for 24 h. In immunoblotting, RAME, not RA, inhibited phosphorylation of S6 (Fig. 2e). These in vitro and in vivo data indicated that methyl residue in RAME caused S6K1 inhibitory effects different from those of RA and 80 μM was the optimal concentration of RAME to fully inhibit S6K1 activity.

**Fig. 3:**
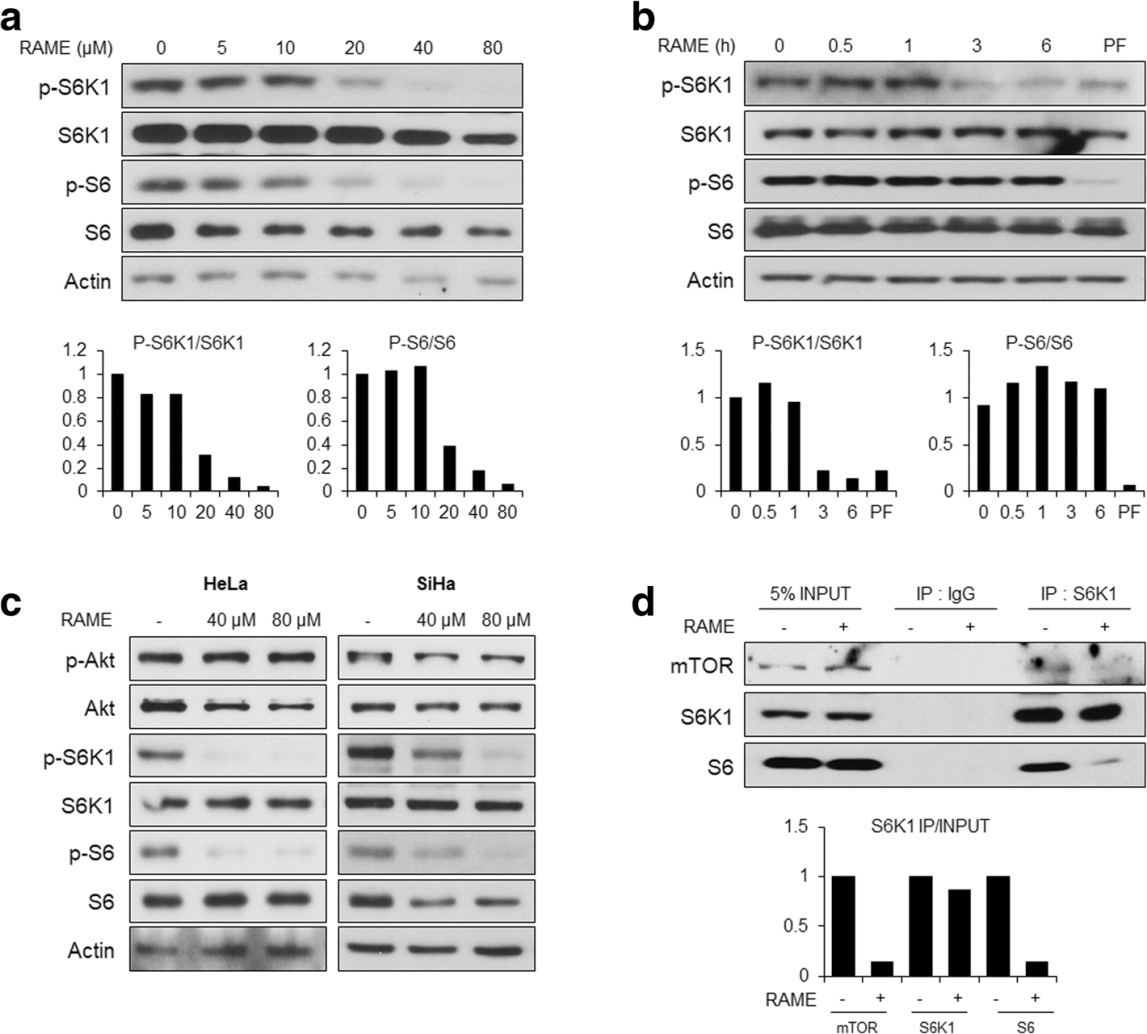
RAME inhibits S6K1 signaling by blocking interaction with mTOR. **a** Immunoblotting analysis and quantification graphs of HeLa cells treated with each concentration of RAME for 24 h. **b** Immunoblotting analysis and quantification graphs of HeLa cells treated with RAME (80 μM) for each time. **c** Immunoblotting analysis of HeLa (left) and SiHa (right) cells treated with RAME (40, 80 μM) for 24 h. **d** Co-IP analysis and a quantification graph using an anti-IgG and S6K1 antibody in DMSO and RAME treated HeLa cells

Similarly, RAME treatment for 24 h dose-dependently reduced phosphorylation of S6 in cervical and lung cancer cells (Fig. 3a; Additional file 1: Figure S3). However, acute treatment with RAME did not show inhibitory effects on S6 phosphorylation, despite declined phosphorylated S6K1 (Fig. 3b). A prior study demonstrated that PF-4708671 inhibited S6K1 activity, but stimulated S6K1 phosphorylation, which was dependent upon mTORC1 [28]. Unlike PF-4708671, RAME decreased the mTOR-dependent phosphorylation of S6K1 T389 in a dose-dependent manner (Fig. 3a, c). mTOR is an enzymatic subunit of both mTORC1 and mTORC2. To investigate the effect of RAME on the enzymatic activity of mTOR, we assessed the phosphorylation of Akt, a substrate of mTORC2, after RAME treatment. Unlike that of S6K1, phosphorylation of Akt was not affected by RAME (Fig. 3c), whereas PF-4708671 increased the level of phosphorylated Akt (Additional file 1: Figure S4). Given that mTOR interacts with and phosphorylates S6K1, we performed a co-immunoprecipitation assay to determine whether the association between mTOR and S6K1 is interrupted by RAME. RAME inhibited S6K1 from interacting with mTOR and S6 (Fig. 3d). These data indicate that RAME effectively inhibits phosphorylation of S6K1 and S6 by blocking the interaction between S6K1 and mTOR.

### RAME induces autophagy in cervical cancer cells

**Fig. 4:**
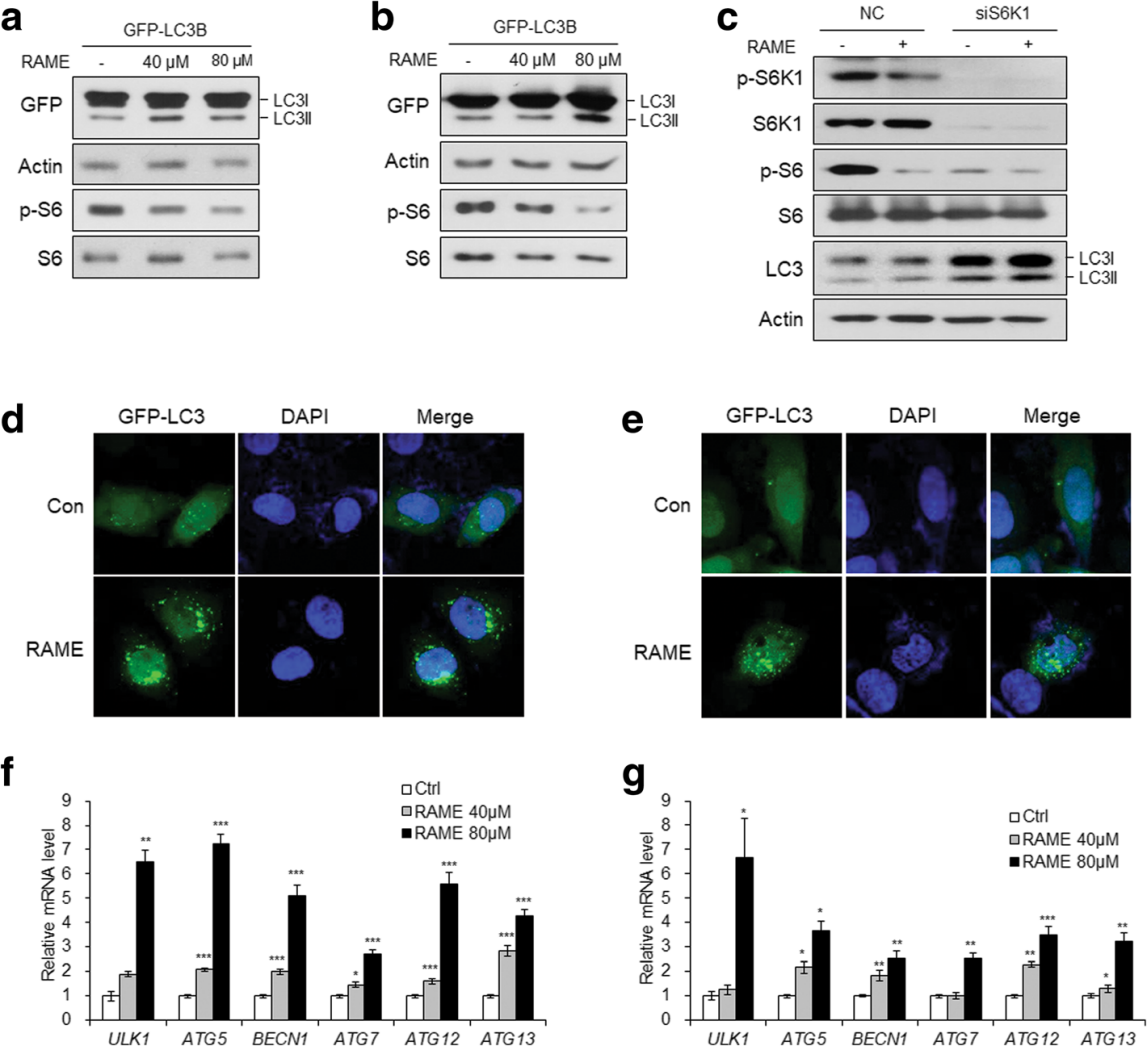
RAME induces autophagy in cervical cancer cells. **a** and **b** Immunoblotting analysis of GFP-LC3B-expressing HeLa (**a**) and SiHa (**b**) cells treated with RAME (40 or 80 μM) for 24 h. **c** Immunoblotting analysis of HeLa cells transfected with siRNA targeting S6K1 and treated with RAME (80 μM) for 24 h. (**d** and **e**) Fluorescent imaging of GFP-LC3B-expressing HeLa (**d**) and SiHa (**e**) cells treated with RAME (80 μM) for 24 h. **f** and **g** The mRNA levels of autophagy-related genes in HeLa (**f**) and SiHa (**g**) cells treated with RAME (40 or 80 μM) for 24 h. Error bars correspond to mean ± SEM (*n* = 3). *p < 0.05, **p < 0.01, ***p < 0.001; unpaired t test

Autophagy is induced during stress or nutrient deprivation states. Through autophagy, the cell facilitates the degradation of damaged cellular components and obtains molecular building blocks and energy [29]. The mTOR/S6K1 pathway is a central regulator of cell growth and proliferation. Additionally, several studies have shown that mTOR and S6K1 inhibits autophagy [30, 31]. The enhancement of a microtubule associated protein light chain 3 (LC3) family members is a marker of cell autophagy activation [32]. Autophagic activity is measured by the conversion of non-lipidated LC3-I to lipidated LC3-II [33]. To examine the effect of RAME on the autophagic process, cervical cancer cell lines (HeLa and SiHa) were transfected with GFP-LC3 and treated with RAME for 24 h. LC3-I and LC3-II were detected using GFP antibody and immunoblotting data showed that treatment with RAME resulted in an increase in lipidated LC3-II in HeLa and SiHa cells (Fig. 4a, b). Endogenous LC3-II was elevated by RAME treatment and knockdown of S6K1, but the effects of RAME did not appear in S6K1-knockdown cells (Fig. 4c), showing that the lipidation of LC3 upon RAME treatment was mediated by S6K1 inhibition. We also observed the fluorescence signal from GFP-LC3 with a confocal microscope and found that LC3 puncta in autophagosomes were formed in HeLa and SiHa cells after treatment with RAME (80 μM) for 24 h (Fig. 4d, e). Recent studies indicated that the transcriptional regulation of autophagy related genes is pivotal for autophagy. For example, the level of Atg8 determines autophagosome size [34] while that of Atg9 is proportional to their number [35], and the amount of Atg7 correlates with autophagy amplitude [36]. RAME treatment increased the mRNA levels of ATG genes (*ULK1*, *ATG5*, *BECN1*, *ATG7*, *ATG12*, and *ATG13*) dose dependently in cervical cancer cells (Fig. 4f, g). Taken together, these results indicate that RAME induces autophagy in cervical cancer cells.

### RAME induces apoptosis in cervical cancer cells

**Fig. 5:**
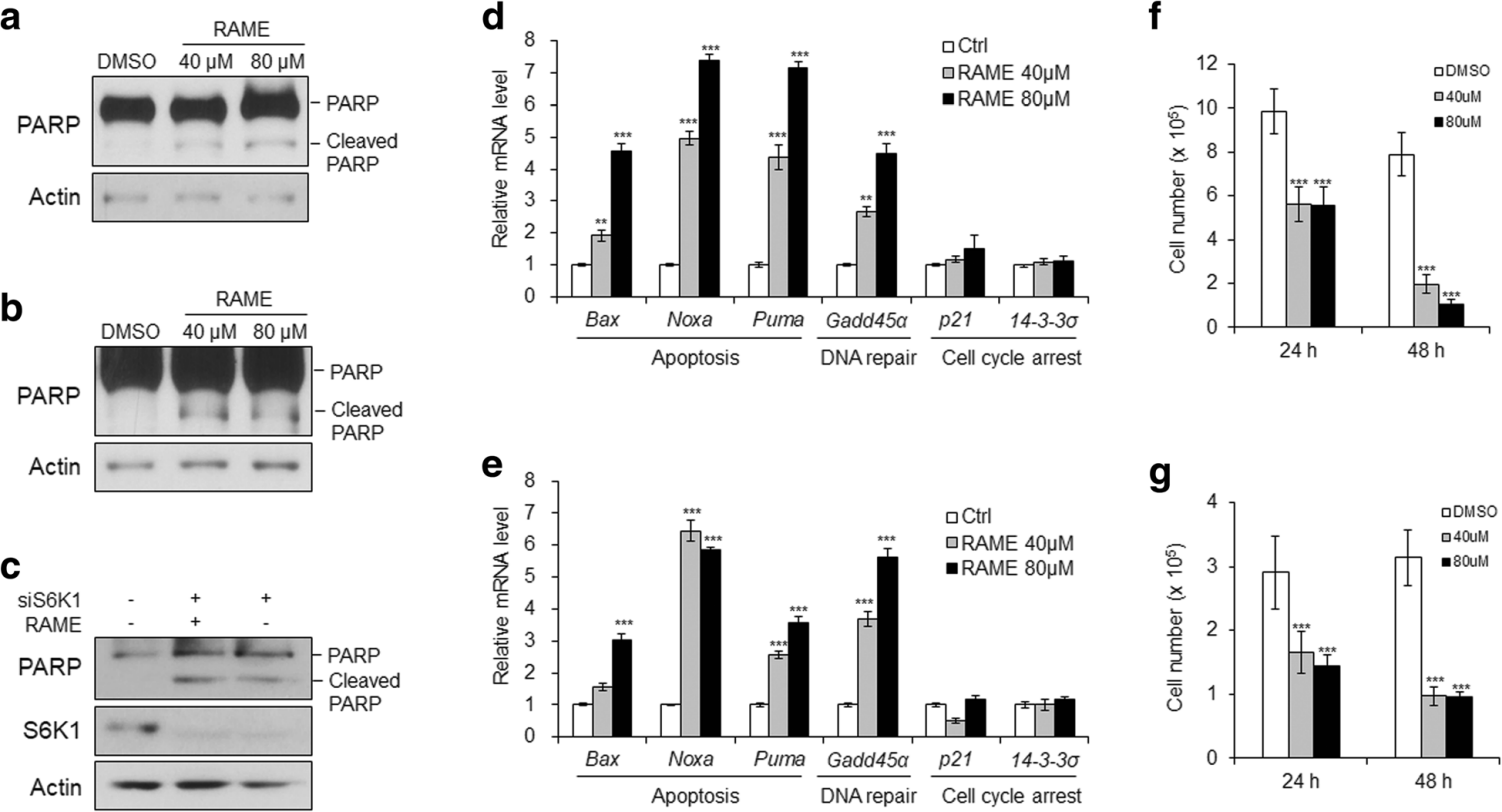
RAME induces apoptosis in cervical cancer cells. **a** and **b** Immunoblotting analysis of HeLa (**a**) and SiHa (**b**) cells treated with RAME (40 or 80 μM) for 24 h. (**c**) Immunoblotting analysis of HeLa cells transfected with siRNA targeting S6K1 and treated with RAME (80 μM) for 24 h. **d** and **e** The mRNA levels of apoptosis, DNA repair, and cell cycle arrest marker genes in HeLa (**d**) and SiHa (**e**) cells treated with RAME (40 or 80 μM) for 24 h. **f** and **g** Cell viability of HeLa (**f**) and SiHa (g) cells treated with RAME (40 or 80 μM) for 24 and 48 h. Error bars correspond to mean ± SEM (*n* = 3). *p < 0.05, **p < 0.01, ***p < 0.001; unpaired t test

As suppressing the phosphorylation of S6K1 induces autophagic cell death [37], we examined the effect of RAME on apoptosis in HeLa and SiHa cells by detecting PARP-1 cleavage. The cleaved forms of PARP-1 were elevated in RAME-treated cervical cancer cells (Fig. 5a, b), which did not increase by RAME in S6K1-deficient cells (Fig. 5c). We also assessed the expression of a variety of tumor-suppressor genes that are associated with apoptosis (*Bax*, *Noxa*, and *Puma*), DNA repair (*Gadd45α*), or cell cycle arrest (*p21* and *14–3-3α*). Treatment of cervical cancer cells with RAME induced transcription of apoptosis-related genes (*Bax*, *Noxa*, and *Puma*) and DNA repair gene (*Gadd45α*), whereas the mRNA levels of the cell cycle arrest genes (*p21* and *14–3-3α*) were not altered by RAME treatment (Fig. 5d, e). We also found that RAME significantly arrested the proliferation of both HeLa and SiHa cells as shown by measuring cell viability (Fig. 5f, g). Moreover, RAME treatment to HeLa cells upregulated the level of p53 level (Additional file 1: Figure S5A), resulting in the increase in apoptotic cell population (Additional file 1: Figure S5B). These results demonstrate that RAME induces apoptotic cell death by exerting an anti-proliferative effect.

### RAME enhances the effects of cisplatin in cervical cancer cells

**Fig. 6:**
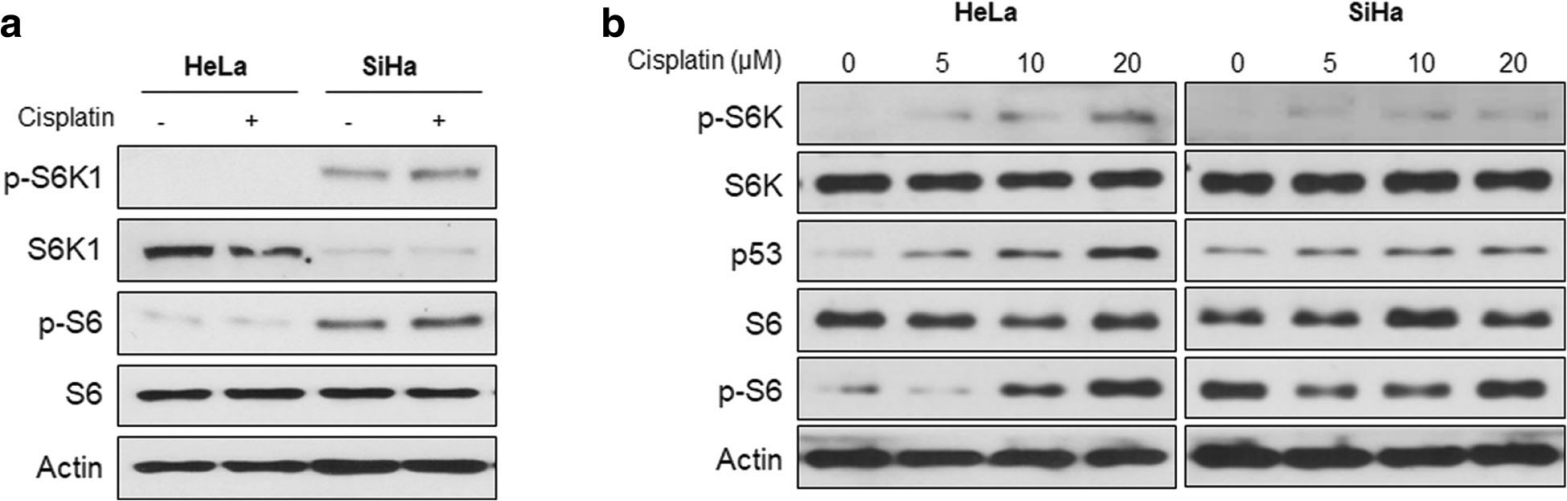
S6K1 is activated in cisplatin-resistant cervical cancer cells. **a** Immunoblotting analysis of HeLa and SiHa cells treated with cisplatin (5 μM) for 24 h. **b** Immunoblotting analysis of HeLa and SiHa cells treated with cisplatin (0, 5, 10, 20 μM) for 24 h

**Fig. 7:**
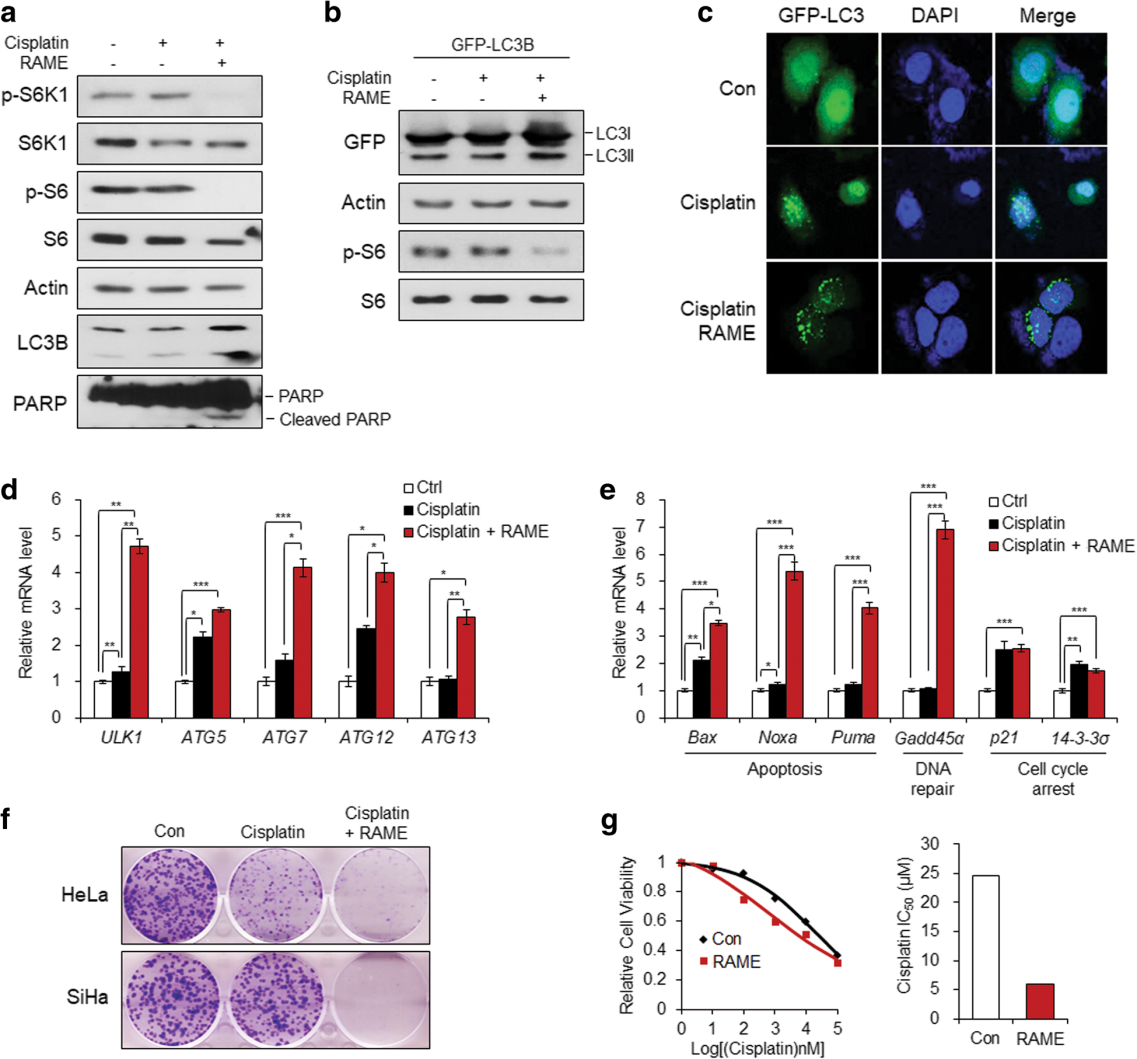
RAME enhances the effects of cisplatin in cervical cancer cells. **a** Immunoblotting analysis of SiHa cells treated with or without cisplatin (5 μM) and RAME (80 μM) for 24 h. **b** Immunoblotting analysis of GFP-LC3B-expressing SiHa cells treated with or without cisplatin (5 μM) and RAME (80 μM) for 24 h. **c** Fluorescent imaging of GFP-LC3B-expressing SiHa cells treated with or without cisplatin (5 μM) and RAME (80 μM) for 24 h. **d** The mRNA levels of autophagy-related genes in SiHa cells treated with or without cisplatin (5 μM) and RAME (80 μM) for 24 h. **e** The mRNA levels of apoptosis, DNA repair, and cell cycle arrest marker genes in SiHa cells treated with or without cisplatin (5 μM) and RAME (80 μM) for 24 h. **f** Clonogenic assay of HeLa and SiHa cells treated with or without cisplatin (1 μM) and RAME (40 μM) for 10~20 days. **g** IC_50_ values of cisplatin in SiHa cells treated with or without RAME (80 μM) for 24 h. Error bars correspond to mean ± SEM (n = 3). *p < 0.05, **p < 0.01, ***p < 0.001; unpaired t test

Cisplatin resistance is the biggest barrier to the successful treatment of cervical cancer [38]. Recent studies suggest that inhibiting the mTOR pathway overcome cisplatin resistance in several types of tumors [39–41]. As SiHa cells are less sensitive to cisplatin than HeLa cells [42], we compared the activation states of S6K1 and its downstream target, S6, in the two cell lines. The basal levels of phosphorylated S6K1 and S6 were higher in SiHa than those in HeLa cells (Fig. 6a). After cisplatin treatment, phosphorylation of S6K1 was dose-dependently increased in HeLa cells (Fig. 6b, left), whereas there was not much change in activation of S6K1 and S6 in SiHa cells (Fig. 6b, right). Therefore, we examined whether inhibition of S6K1 with RAME caused an increase in sensitivity to cisplatin. Treatment of SiHa cells with RAME ablated phosphorylation of both S6K1 and S6 also in the presence of cisplatin (Fig. 7a). Because the inhibition of S6K1 induced autophagy in cervical cancer, we investigated whether co-treatment with cisplatin and RAME induces autophagy more effectively than cisplatin alone. An immunoblotting assay with GFP-LC3B transfected SiHa cells showed that GFP-LC3-II, a lapidated form, increased more after co-treatment with cisplatin and RAME (Fig. 7b), which was also observed in endogenous LC3-II (Fig. 7a). The confocal microscopic image showed that the formation of the autophagosome was more detected after co-treatment with cisplatin and RAME in SiHa cells (Fig. 7c). Moreover, the transcription of autophagy-related genes was dramatically elevated after dual treatment compared to treatment with cisplatin alone (Fig. 7d), implying that combined treatment with cisplatin and RAME augmented autophagy in cisplatin resistant SiHa cells. Next, to confirm that RAME induces apoptosis after combined treatment, we assessed the expression of apoptotic genes. Consistent with the increase in autophagy, the mRNA levels of apoptosis related genes (*Bax*, *Noxa*, and *Puma*) and a DNA repair gene (*Gadd45a*) significantly increased after combination treatment (Fig. 7e). Treatment with RA, the parent compound of RAME, however, did not result in enhancing the expression of autophagy-related genes (Additional file 1: Figure S6A) or apoptotic genes (Additional file 1: Fig. S6B) when used in combination with cisplatin. Interestingly, cell cycle arrest genes (*p21* and *14–3-3α*) increased only after RA treatment, but not after RAME treatment (Fig. 7e; Additional file 1: Figure S6B). The apoptotic marker, cleaved PARP-1, also increased after combination treatment (Fig. 7a). Moreover, clonogenic assay data showed that co-treatment with RAME enhanced the inhibitory effects of cisplatin against colony formation in both HeLa and SiHa cells (Fig. 7f). Lastly, we measured the cell viability of cisplatin-treated SiHa cells by co-treating with RAME at different concentrations. The IC_50_ values of cisplatin to block the survival of cervical cancer cells markedly decreased after RAME treatment (Fig. 7g). Collectively, these data imply that RAME enhances the effects of cisplatin in cervical cancer cells.

## Discussion

In this study, we reveal that a natural compound rosmarinic acid methyl ester (RAME) exerts anti-cancer effects against cervical cancer by inhibiting mTOR/S6K1 pathway. The structure-based computational approach led to the identification of several small molecules, including RAME, which were expected to target S6K1. Successively through cell-based assays, we found that RAME effectively inhibits the activation of S6K1 by mTOR, whereas rosmarinic acid cannot affect mTOR/S6K1 signaling pathway. Rosmarinic acid (RA) is a natural polyphenolic substance found in various Lamiaceae herbs such as perilla [43], rosemary [44], sage [45], mint [46], basil [47], and thyme [48]. A number of studies have reported the biological effects of RA and one of its derivatives RAME, including anti-inflammatory [49, 50], anti-allergic [51, 52], and anti-microbial [53] effects. Additionally, here we evaluated the anti-tumor effects and mechanisms of action of RAME that were not observed after RA treatment (Additional file 1: Figure S6). Interestingly, there are several medicinal chemistry data demonstrating that the length of the alkyl side chain determines the bioactivity of RA derivatives [52–54]. According to our virtual screening data in Fig. 1b, methyl ester group of RAME enters the groove around Met225 of S6K1 properly, which is advantageous for VDW interaction.

mTOR pathway is a master regulator of cell growth/size and protein synthesis that can lead to tumorigenesis [55]. Therefore, pharmacological inhibition of the mTOR signaling pathway is emerging as a useful therapeutic strategy for various cancers [56]. Several recent studies have shown that treatment with rapamycin, the most established mTOR/S6K1 inhibitor, induces autophagy and apoptotic cell death in cervical cancer cells as well as synergistic therapeutic responses in combination with cisplatin [9, 12, 22]. However, chronic use of rapamycin was found to cause unexpected insulin resistance [57, 58], which was mediated by impaired activation of the mTORC2/Akt pathway [59, 60]. Conversely, it was also reported that rapamycin enhances activation of Akt through a negative feedback loop [61]. These adverse effects of rapamycin necessitated the development of more specific mTORC1 inhibitors. As displayed in Fig. 3c and Additional file 1: Figure S4, RAME treatment for 24 h did not much alter the phosphorylation of Akt, suggesting that clinical and chronic use of RAME would provide more benefits and avoid the side effects on glucose homeostasis.

Occurrence of cisplatin resistance is a widespread phenomenon in cancer patients who have undergone platinum-based chemotherapy. Cancer cells that acquire cisplatin resistance lack apoptotic capacity with frequently observed abnormal activation of the Akt/mTOR pathway [19]. Our data also show that the treatment of cervical cancer cells with cisplatin induced activation of S6K1 and S6 (Fig. 6b), whose levels were already high in the cisplatin-resistant cervical cancer cell line (Fig. 6a). RAME treatment combined with cisplatin sensitized the resistant cells, reducing the IC_50_ value of cisplatin and promoting autophagy and apoptosis (Fig. 7g). Therefore, combining RAME with cisplatin can overcome tolerance and the adverse effects of high doses of cisplatin alone.

Cisplatin sensitivity is enhanced by co-treatment with mTOR inhibitors in various other cancers, including ovarian cancer [18, 19], lung cancer [20], and osteosarcoma [21, 24], as well as cervical cancer. The inactivation of S6K1 by treatment with RAME also occurred in the non-small cell lung cancer cell lines, A549 and H1299, though the effect was less in the cisplatin-resistant cell line, H1299 (Additional file 1: Figure S3). Further studies to explore the anti-tumor activity of RAME in lung cancer would broaden the applicable therapeutic range of RAME.

## Conclusions

In summary, we elucidate the therapeutic potential of a newly found mTOR/S6K1 inhibitor, RAME, for the treatment of cervical cancer patients. Although the conventional mTOR inhibitor inevitably caused unpleasant side effects because of the additional inhibition of Akt, we here present that RAME specifically blocks the mTORC1/S6K1 signaling pathway without extra inhibition of Akt. Consequently, RAME induced the overexpression of multiple factors implicated in autophagy and apoptosis, leading to suppression of cell proliferation. Therefore, our findings suggest that RAME can be used as a promising anticancer agent for the treatment of cervical cancer, even when possessing chemoresistance.

## Additional file

Additional file 1: **Figure S1**. Chemical structures of high-ranking virtual screening hits from both docking- and similarity-based method. **Figure S2.** RAME inhibits H2B phosphorylation by S6K1 in vitro. In vitro kinase assay with RAME was performed in a dose dependent manner using recombinant H2B, active S6K1, and cold-ATP. **Figure S3.** Effects of RAME on lung cancer cell lines. Immunoblotting analysis of A549 and H1299 cells treated with RAME (40, 80 μM) for 24 h. **Figure S4.** Effects of RAME and PF-4708671 on phosphorylation of Akt. Immunoblotting analysis of HeLa cells treated with RAME (40 μM) or PF-4708671 (20 μM) for 24 h. **Figure S5.** RAME induces apoptosis in cervical cancer cells. (A) Immunoblotting analysis of HeLa cells treated with RAME (40 or 80 μM) for 24 h. (B) Flow cytometric analysis of HeLa cells treated with RAME (80 μM) for 24 h. **Figure S6.** RA does not enhance the effects of cisplatin in cervical cancer cells. (A) The mRNA levels of autophagy-related genes in SiHa cells treated with or without cisplatin (5 μM) and RA (80 μM) for 24 h. (B) The mRNA levels of apoptosis, DNA repair, and cell cycle arrest marker genes in SiHa cells treated with or without cisplatin (5 μM) and RA (80 μM) for 24 h. Error bars correspond to mean ± SEM (*n* = 3). *p < 0.05, **p < 0.01, ***p < 0.001; unpaired t test. (PPTX 1004 kb)

## Data Availability

All data generated or analyzed during this study are reflected in the present published article and its supplementary information files.

## Abbreviations

GFP: Green fluorescent protein
HPV: Human papillomavirus
IC_50_: The half maximal inhibitory concentration
LC3: Microtubule-associated protein 1A/1B-light chain 3
mTOR: Mammalian target of rapamycin
PARP: Poly (ADP-ribose) polymerase
RA: Rosmarinic acid
RAME: Rosmarinic acid methyl ester
S6K1: Ribosomal protein S6 kinase 1
