# Supplementary material for: Identification of a novel S6K1 inhibitor, rosmarinic acid methyl ester, for treating cisplatin-resistant cervical cancer
Source: BMC Cancer. 2019 Aug 6;19:773. doi: 10.1186/s12885-019-5997-2 (PMC6683399; doi:10.1186/s12885-019-5997-2)
Supplement: Supplementary file 1 — Figure S1. Chemical structures of high-ranking virtual screening hits from both docking- and similarity-based method. Figure S2. RAME inhibits H2B phosphorylation by S6K1 in vitro. In vitro kinase assay with RAME was performed in a dose dependent manner using recombinant H2B, active S6K1, and cold-ATP. Figure S3. Effects of RAME on lung cancer cell lines. Immunoblotting analysis of A549 and H1299 cells treated with RAME (40, 80 μM) for 24 h. Figure S4. Effects of RAME and PF-4708671 on phosphorylation of Akt. Immunoblotting analysis of HeLa cells treated with RAME (40 μM) or PF-4708671 (20 μM) for 24 h. Figure S5. RAME induces apoptosis in cervical cancer cells. (A) Immunoblotting analysis of HeLa cells treated with RAME (40 or 80 μM) for 24 h. (B) Flow cytometric analysis of HeLa cells treated with RAME (80 μM) for 24 h. Figure S6. RA does not enhance the effects of cisplatin in cervical cancer cells. (A) The mRNA levels of autophagy-related genes in SiHa cells treated with or without cisplatin (5 μM) and RA (80 μM) for 24 h. (B) The mRNA levels of apoptosis, DNA repair, and cell cycle arrest marker genes in SiHa cells treated with or without cisplatin (5 μM) and RA (80 μM) for 24 h. Error bars correspond to mean ± SEM (n = 3). *p < 0.05, **p < 0.01, ***p < 0.001; unpaired t test. (PPTX 1004 kb) [file 12885_2019_5997_MOESM1_ESM.pptx]

## Slide 1
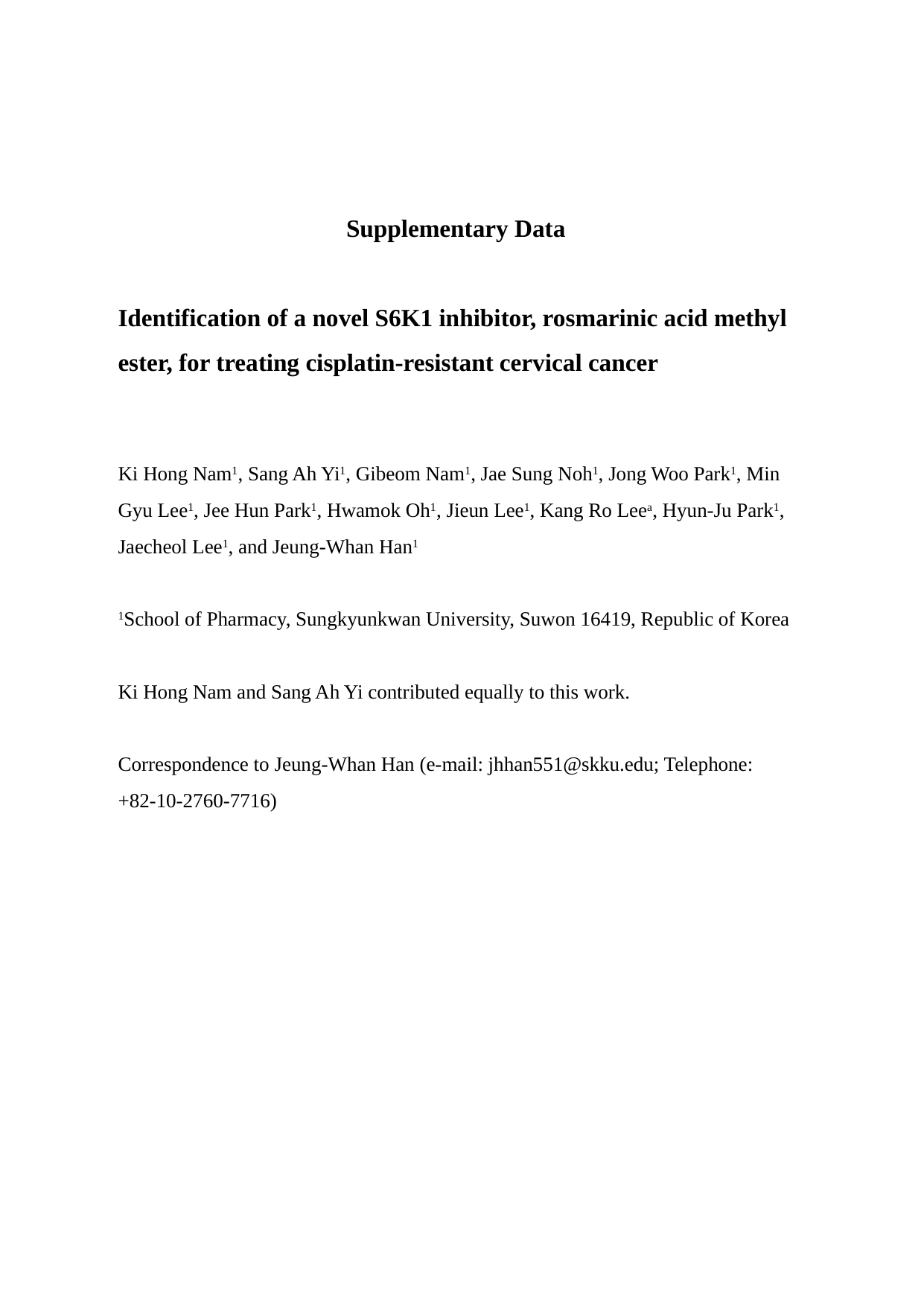

Supplementary Data
Identification of a novel S6K1 inhibitor, rosmarinic acid methyl ester, for treating cisplatin-resistant cervical cancer
Ki Hong Nam1, Sang Ah Yi1, Gibeom Nam1, Jae Sung Noh1, Jong Woo Park1, Min Gyu Lee1, Jee Hun Park1, Hwamok Oh1, Jieun Lee1, Kang Ro Leea, Hyun-Ju Park1, Jaecheol Lee1, and Jeung-Whan Han1
1School of Pharmacy, Sungkyunkwan University, Suwon 16419, Republic of Korea
Ki Hong Nam and Sang Ah Yi contributed equally to this work.
Correspondence to Jeung-Whan Han (e-mail: jhhan551@skku.edu; Telephone: +82-10-2760-7716)

## Slide 2
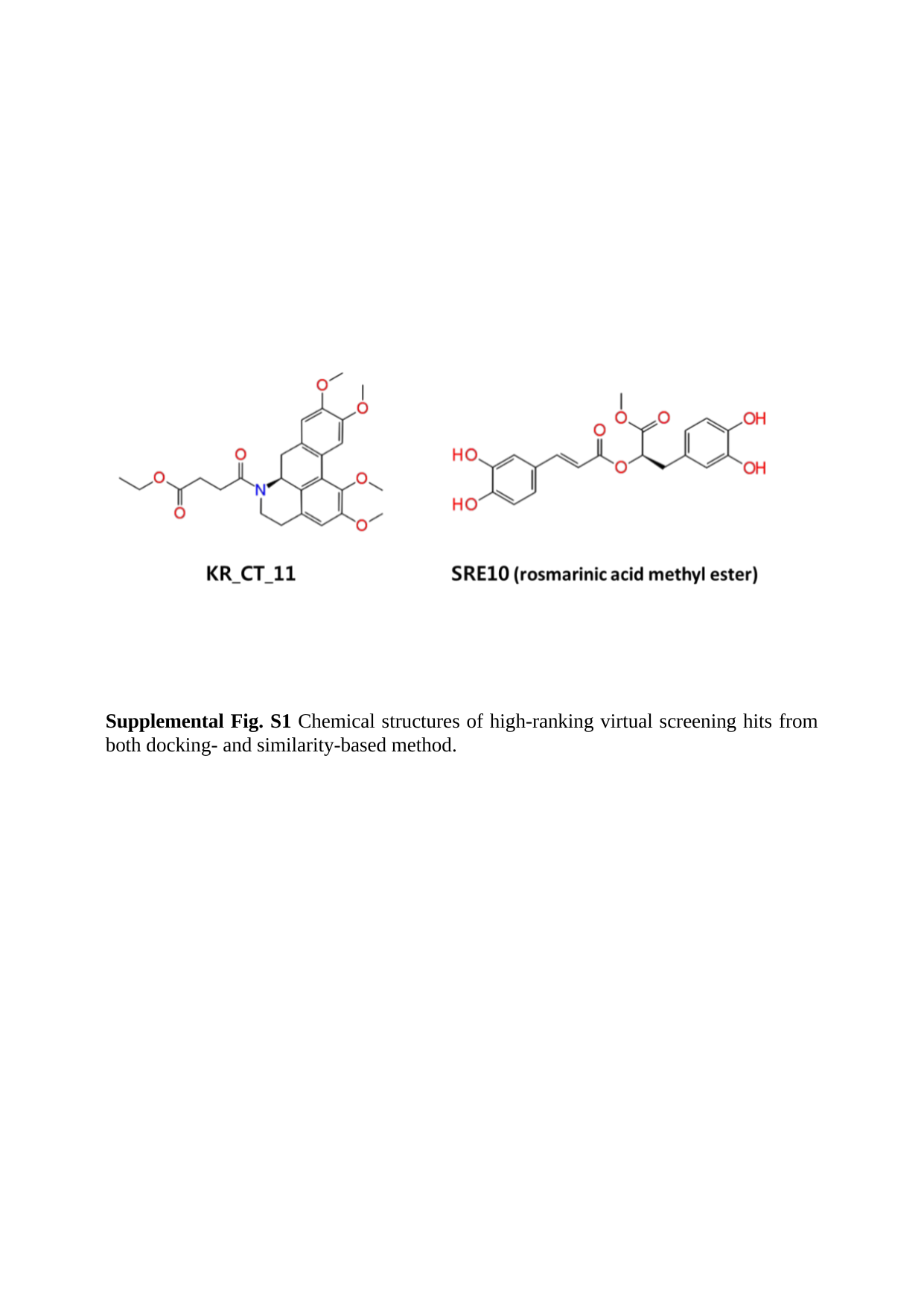

Supplemental Fig. S1 Chemical structures of high-ranking virtual screening hits from both docking- and similarity-based method.

## Slide 3
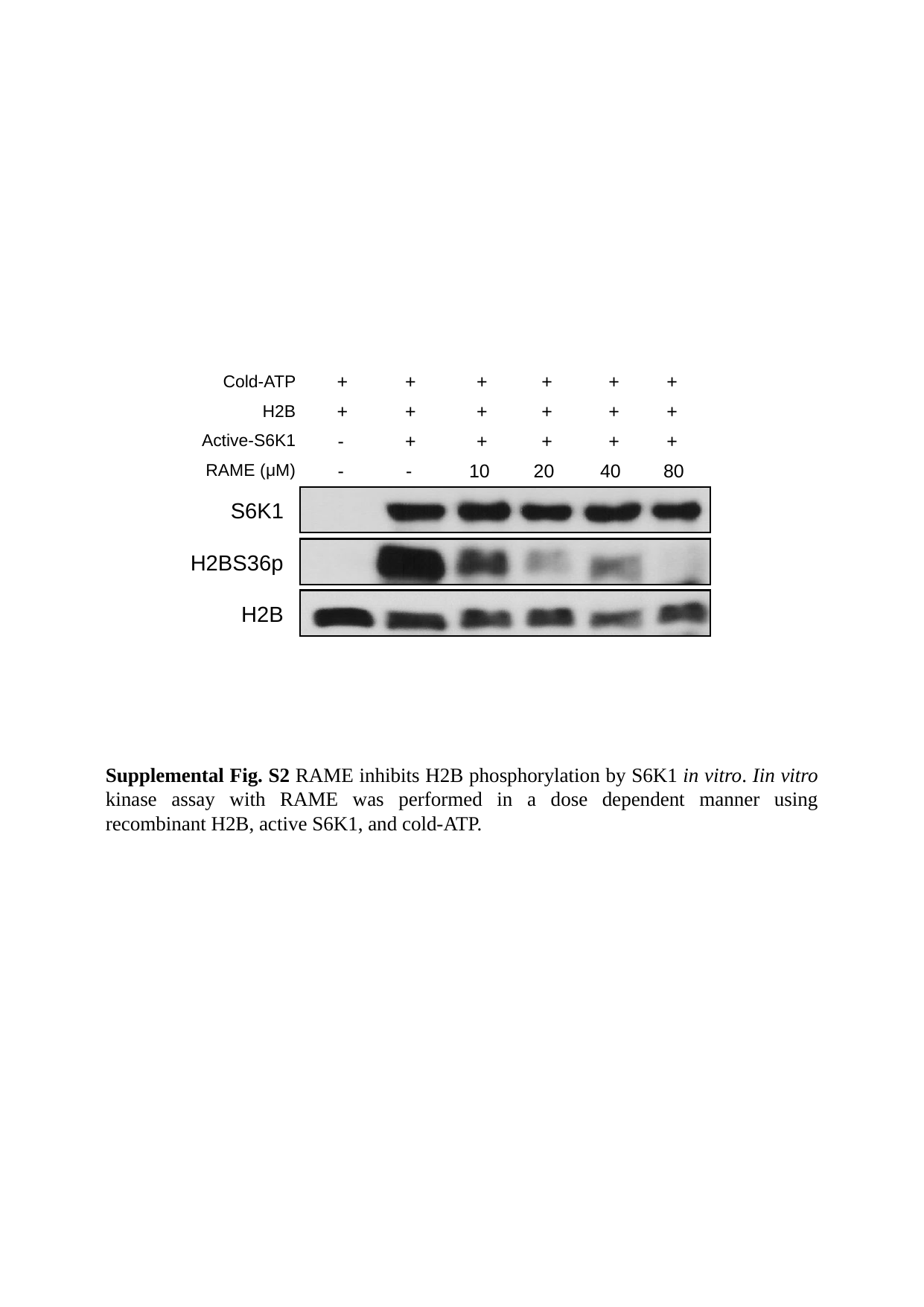

+
+
+
+
+
+
Cold-ATP
+
+
+
+
+
+
H2B
-
+
+
+
+
+
Active-S6K1
RAME (μM)
-
-
10
20
40
80
S6K1
H2BS36p
H2B
Supplemental Fig. S2 RAME inhibits H2B phosphorylation by S6K1 in vitro. Iin vitro kinase assay with RAME was performed in a dose dependent manner using recombinant H2B, active S6K1, and cold-ATP.

## Slide 4
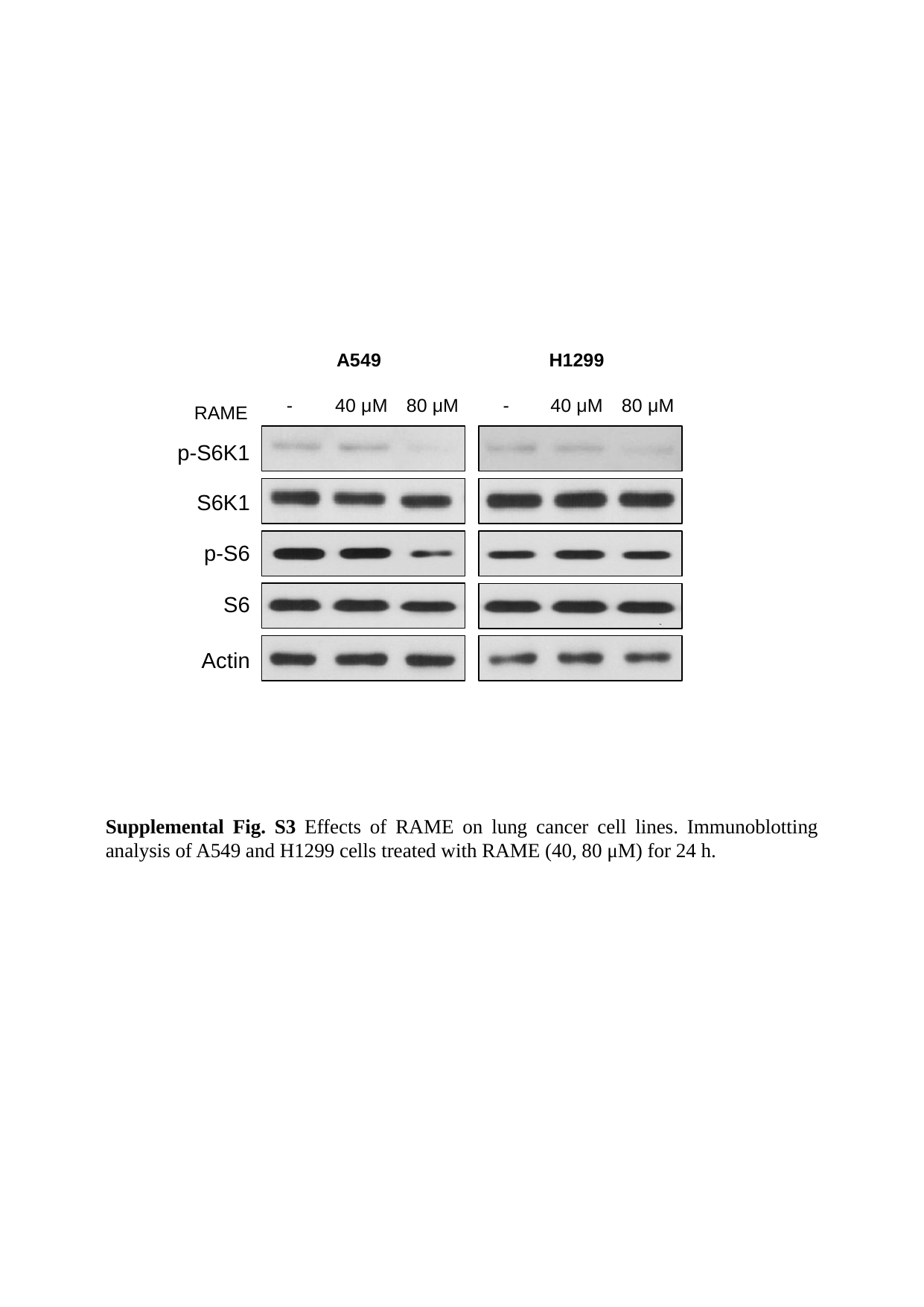

A549
H1299
-
40 μM
80 μM
-
40 μM
80 μM
RAME
p-S6K1
S6K1
p-S6
S6
Actin
Supplemental Fig. S3 Effects of RAME on lung cancer cell lines. Immunoblotting analysis of A549 and H1299 cells treated with RAME (40, 80 μM) for 24 h.

## Slide 5
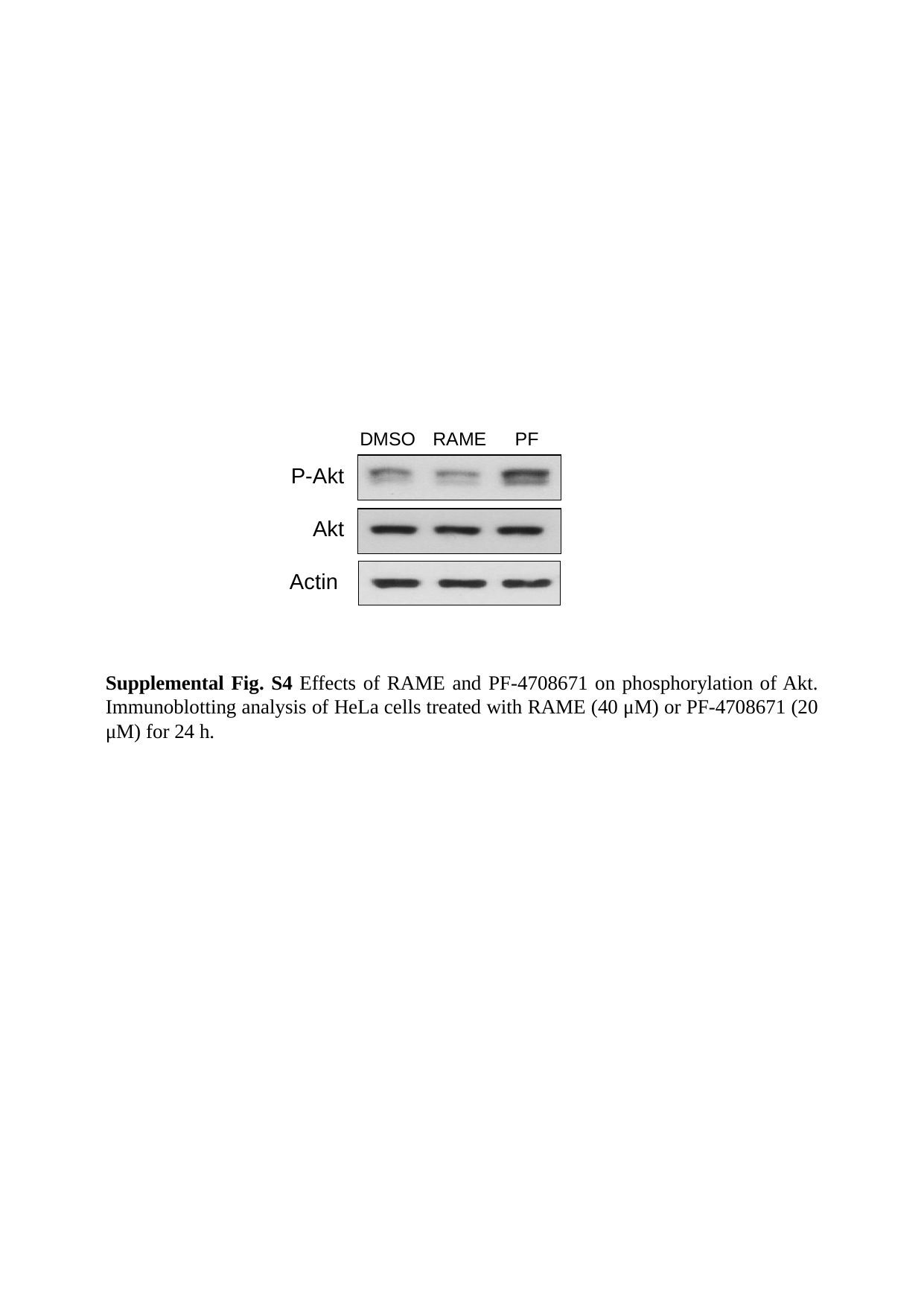

DMSO
RAME
PF
P-Akt
Akt
Actin
Supplemental Fig. S4 Effects of RAME and PF-4708671 on phosphorylation of Akt. Immunoblotting analysis of HeLa cells treated with RAME (40 μM) or PF-4708671 (20 μM) for 24 h.

## Slide 6
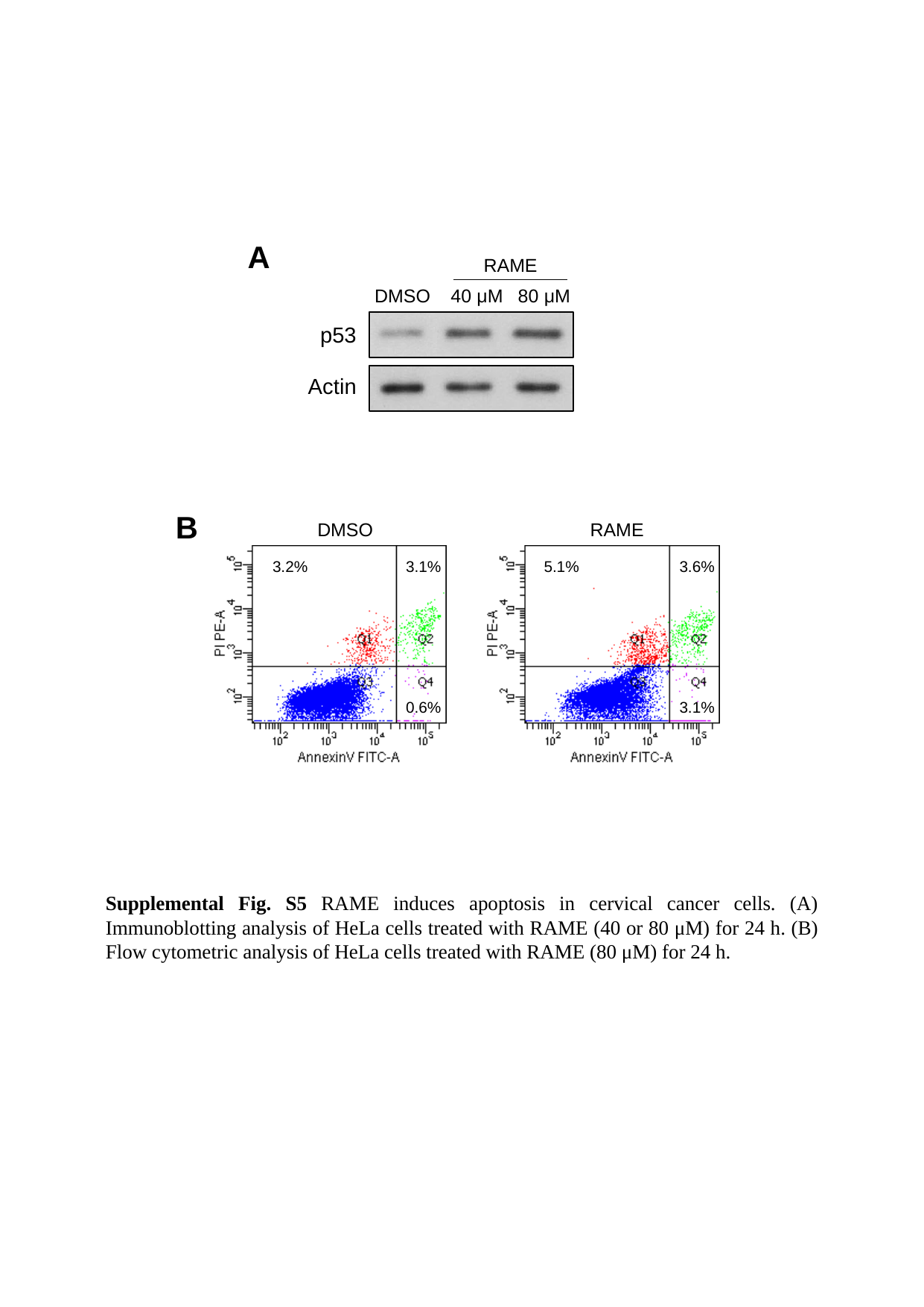

A
RAME
DMSO
40 μM
80 μM
p53
Actin
B
DMSO
RAME
3.2%
3.1%
5.1%
3.6%
0.6%
3.1%
Supplemental Fig. S5 RAME induces apoptosis in cervical cancer cells. (A) Immunoblotting analysis of HeLa cells treated with RAME (40 or 80 μM) for 24 h. (B) Flow cytometric analysis of HeLa cells treated with RAME (80 μM) for 24 h.

## Slide 7
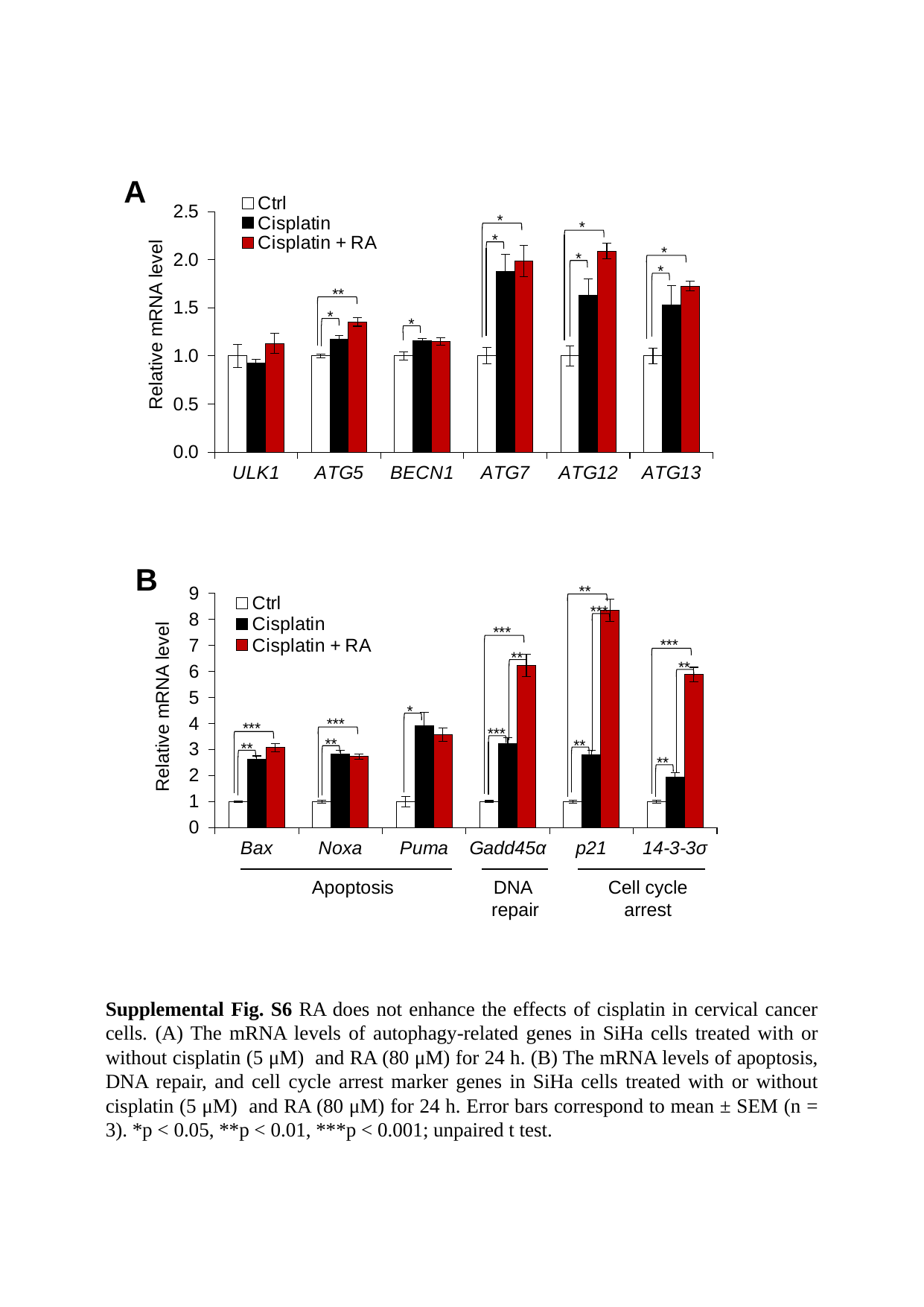

A
### Chart
| Category | | | |
|---|---|---|---|
| ULK1 | 1.0 | 0.924568027241431 | 1.12837737201517 |
| ATG5 | 1.0 | 1.17401151378295 | 1.35465088033503 |
| BECN1 | 1.0 | 1.16119331634356 | 1.15104998199953 |
| ATG7 | 1.0 | 1.8745365210914 | 1.98347559639868 |
| ATG12 | 1.0 | 1.62700634655772 | 2.08885359386688 |
| ATG13 | 1.0 | 1.528160383438 | 1.72516044703278 |*
*
*
*
*
*
**
*
*
Relative mRNA level
B
### Chart
| Category | | | |
|---|---|---|---|
| Bax | 1.0 | 2.61596611943366 | 3.075451819277 |
| Noxa | 1.0 | 2.83116636003017 | 2.73430505019073 |
| Puma | 1.0 | 3.9159835835218 | 3.57344111938509 |
| Gadd45α | 1.0 | 3.22179656205239 | 6.2397471674526 |
| p21 | 1.0 | 2.79414508859916 | 8.34981894078446 |
| 14-3-3σ | 1.0 | 1.93215835345927 | 5.88207068691228 |**
***
***
***
**
**
Relative mRNA level
*
***
***
***
**
**
**
**
Apoptosis
DNA
repair
Cell cycle
arrest
Supplemental Fig. S6 RA does not enhance the effects of cisplatin in cervical cancer cells. (A) The mRNA levels of autophagy-related genes in SiHa cells treated with or without cisplatin (5 μM) and RA (80 μM) for 24 h. (B) The mRNA levels of apoptosis, DNA repair, and cell cycle arrest marker genes in SiHa cells treated with or without cisplatin (5 μM) and RA (80 μM) for 24 h. Error bars correspond to mean ± SEM (n = 3). *p < 0.05, **p < 0.01, ***p < 0.001; unpaired t test.
